# Diversity Partitioning of Stony Corals Across Multiple Spatial Scales Around Zanzibar Island, Tanzania

**DOI:** 10.1371/journal.pone.0009941

**Published:** 2010-03-29

**Authors:** Assaf Zvuloni, Robert van Woesik, Yossi Loya

**Affiliations:** 1 Department of Zoology, Tel Aviv University, Ramat Aviv, Tel Aviv, Israel; 2 H. Steinitz Marine Biology Laboratory, Interuniversity Institute for Marine Sciences of Eilat, Eilat, Israel; 3 Department of Biological Sciences, Florida Institute of Technology, Melbourne, Florida, United States of America; University of Pretoria, South Africa

## Abstract

**Background:**

The coral reefs of Zanzibar Island (Unguja, Tanzania) encompass a considerable proportion of the global coral-reef diversity and are representative of the western Indian Ocean region. Unfortunately, these reefs have been recently subjected to local and regional disturbances. The objectives of this study were to determine whether there are potentially non-random processes forcing the observed coral diversity patterns, and highlight where and at which spatial scales these processes might be most influential.

**Methodology/Principal Findings:**

A hierarchical (nested) sampling design was employed across three spatial scales, ranging from transects (≤20 m), stations (<100 m), to sites (<1000 m), to examine coral diversity patterns. Two of the four sites, Chumbe and Mnemba, were located within Marine Protected Areas (MPAs), while the other two sites, Changuu and Bawe, were not protected. Additive partitioning of coral diversity was used to separate regional (total) diversity (*γ*) into local *α* diversity and among-sample *β* diversity components. Individual-based null models were used to identify deviations from random distribution across the three spatial scales. We found that Chumbe and Mnemba had similar diversity components to those predicted by the null models. However, the diversity at Changuu and Bawe was lower than expected at all three spatial scales tested. Consequently, the relative contribution of the among-site diversity component was significantly greater than expected. Applying partitioning analysis for each site separately revealed that the within-transect diversity component in Changuu was significantly lower than the null expectation.

**Conclusions/Significance:**

The non-random outcome of the partitioning analyses helped to identify the among-sites scale (i.e., 10's of kilometers) and the within-transects scale (i.e., a few meters; especially at Changuu) as spatial boundaries within which to examine the processes that may interact and disproportionately differentiate coral diversity. In light of coral community compositions and diversity patterns we strongly recommend that Bawe be declared a MPA.

## Introduction

Biodiversity is not homogeneously distributed across the planet. Understanding how and why diversity changes across multiple-spatial scales is still one of the most challenging tasks facing contemporary ecology in general [1], and tropical-marine ecology in particular [2]–[3]. Elucidating diversity patterns has wide-ranging applications, from identification of appropriate spatial boundaries for studying mechanisms that generate and maintain biodiversity, to predictions of how local and regional environmental changes will affect diversity at different levels of organization. These predictions may involve evolutionary, environmental, and ecological processes interacting at a variety of scales [4]–[5]. In marine ecosystems, while regional diversity allows some insight into local patterns [3], local diversity might be very different from those found over broader scales.

**Figure 1:**
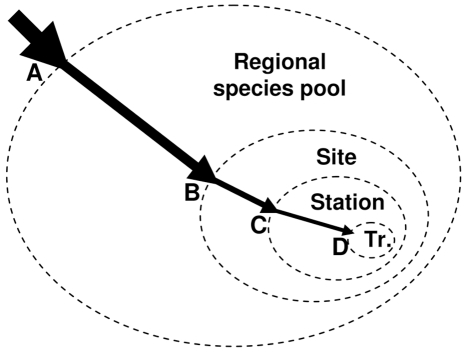
Schematic representation of species passing through a series of scale-dependent “filters” (i.e., processes), which represent historical, environmental and ecological constraints on the arrival and survival of species. This representation is modified from Hillebrand and Blenckner [4] to represent the nested sampling design of the present study. ‘A’ represents the first filter, which determines the regional species pool; filter ‘B’ determines the species composition within a site in the region; filter ‘C’ determines the species composition within a station at the site; and filter ‘D’ determines the species composition within a transect (Tr.) at the station.

Theoretically, the assembly of local communities can be visualized as the result of species passing through a series of “filters” ([4]; Fig. 1). Indeed, these filters may represent diverse processes interacting on multiple spatial scales, which may have direct influences on the arrival and survival of organisms [6]–[7]. There will be some transmission of process signals from one scale to the next, but different processes emerge at different scales [8]. For example, at broad scales (e.g., 10s–100s km) these filters may represent historical and oceanographic constraints (e.g., migration, emigration, regional-scale speciation and gene flow), all of which are intertwined with climate oscillations and glaciation events that change regional-current patterns and population connectivity [9]. At smaller scales these filters may be environmental and ecological (e.g., differential diurnal temperature, irradiance, turbidity, sedimentation, predation, and herbivory; [10]–[11]. However, some driving factors may interact on multiple scales. For example, seasonal-low temperatures clearly prevent most coral species growing at relatively high latitudes, but diurnal temperature extremes also select against temperature-sensitive species on shallow reef flats.

Additive partitioning of species diversity is a promising approach to distinguish among spatial diversity patterns using hierarchical sampling [12]–[14]. Unlike most studies that compare mean diversity among a number of samples, additive partitioning distinguishes the specific contribution of each hierarchical level relative to overall diversity. Comparing observed patterns with those predicted by null models, gives further insight into non-random selective mechanisms that may disproportionately differentiate overall diversity. For example, if diversity is lower than predicted from random at the scale of habitat, then we can safely assume that the habitat conditions are conducive to strong-selective filters, although we do not know precisely what those filters entail, without experimental manipulations. The utility of this analysis has been criticized because the specific processes responsible for the observed patterns in many cases can be difficult to tease apart [15]. However, in a conservation context identifying appropriate spatial boundaries at which these processes interact can be highly informative, even if the underlying processes themselves are not yet evident [16].

The coral reefs of Zanzibar Island (Unguja), Tanzania, support a large proportion of regional reef-coral diversity [17], and are representative reef assemblages of the western Indian Ocean region [18]. However, these reefs are subjected to natural and, more recently, anthropogenic disturbances [18]–[23]. One of the main threats to the reefs is over-exploitation of reef resources and extractive fishing [24]–[25]. Reports of the status of Tanzanian fisheries and the coral reefs in general are grim, because fishing effort has doubled in less than 20 years [25], and destructive methods (e.g., dynamite) are commonplace [26]. In addition, other stressors, such as sedimentation, eutrophication and pollution, add chronic stress to reef-coral communities that in turn force local extirpation and reduce regional coral diversity [22], [27].

Nevertheless, there is a paucity of data describing the composition and spatial diversity of reef-coral assemblages around Zanzibar [25], [28]–[32]. In areas with a high degree of anthropogenic disturbances, conservation strategies must consider the distribution patterns of the organisms within the region [33]–[34]. This conservation vision requires a sophisticated knowledge of how biodiversity is organized across different spatial scales [35].

In a noteworthy study Belmaker *et al.* [36] tested variations in diversity partitioning of coral-dwelling fishes and their hosts in three regions across the Indo-Pacific - Red Sea, Tanzania, and the Great Barrier Reef. In their study, two sites in Zanzibar (Bawe and Chumbe) were sampled within the Tanzanian region. Since Belmaker's study focused on fish diversity within branching-coral heads, only branching corals from the genera *Stylophora*, *Pocillopora*, *Seriatopora* and *Acropora* were sampled.

In this study we focused on stony-coral assemblages. Our hypothesis (to be tested) was that coral assemblages around Zanzibar vary non-randomly across spatial scales. Thus, we sought to quantitatively describe composition and diversity of stony-corals on the Zanzibar reefs and examine their distribution across a hierarchy of spatial scales. Our major objective was to identify where and at which spatial scales non-stochastic processes might interact and have direct influences on the arrival and survival of corals. This would enable us to highlight the appropriate spatial boundaries for studying these processes.

## Methods

### Study sites

**Figure 2:**
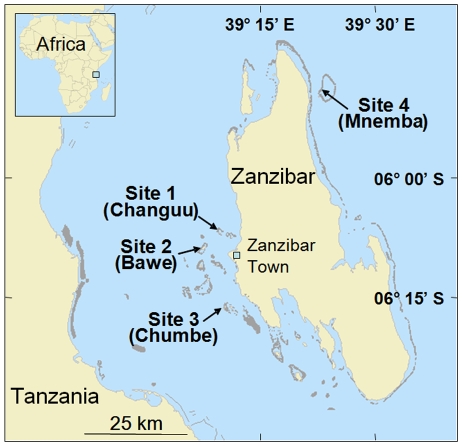
Location of the four study sites around Zanzibar Island, Tanzania. Site 1 (Changuu; 06° 07′ 07.06″ S, 39° 09′ 59.74″ E), Site 2 (Bawe; 06° 08′ 52.03″ S, 39° 08′ 10.09″ E), Site 3 (Chumbe; 06° 16′ 38.57″ S, 39° 10′ 31.06″ E) and Site 4 (Mnemba; 05° 48′ 58.71″ S, 39° 22′ 59.20″ E).

Reef surveys were conducted in November 2006 on four reef sites around Zanzibar (Fig. 2). All four study sites were established in the wave-sheltered shallow reefs habitat, approximately 1–3 m below low water datum, and which experienced relatively little wave action. We focused our sampling effort on one habitat in order to increase spatial resolution of that habitat. Sites 1 and 2 were located 5 and 7 km from Zanzibar Town (respectively). They are both influenced to varying degrees by fishing [26], [29], and receive untreated town and harbor sewage discharge, and are subjected to a number of other destructive activities [37]–[38]. In contrast, the other two sites, Sites 3 and 4, were located within Marine Protected Areas (MPAs). Site 3, located ca. 13 km south of Zanzibar Town, has been a private nature reserve, developed and managed by the Chumbe Island Coral Park (CHICOP), since 1992. Site 4, located ca. 2.5 km off the north-eastern tip of Zanzibar, has been protected from extractive resource use since 1989. Fishing has been strictly banned and all tourist activities are controlled at Sites 3 and 4.

### Coral-community survey

A hierarchical (nested) sampling design was employed to assess three spatial scales of influence: (1) transects (≤20 m), (2) stations (<100 m), and (3) sites (<1000 m). The design consisted of four sampling sites (Fig. 2) that were separated by at least 5 km. At each site, two randomly allocated stations were established, separated by ca. 300 m. Within each station six 20×1 m belt-transects were randomly placed over an area of ca. 75×20 m to sample composition of coral assemblages. Each belt-transect was subdivided into twenty 1×1 m quadrats, which were photographed with a digital camera (Olympus C-5060) attached to a 1×1 m PVC-frame. Field sampling was performed by a single surveyor (AZ) to reduce sampler bias. From the photographs taken in the field, corals were enumerated and identified to the lowest taxonomic resolution. However, since some closely-related species were difficult to differentiate, they were combined and analyzed as taxonomic units (hereafter TAUs). The complete list of TAUs, including the coral species belonging to each TAU, is provided in Table S1 in the electronic supplementary material. To prevent biases related to the boundary effect of the sampling units, only the corals with centers lying within the sampling unit were enumerated (following the “*center rules*” scheme of Zvuloni *et al.* [39]).

### Data analyses

Additive partitioning of coral diversity was used to quantify the spatial distribution of diversity patterns [12], [40]–[41]. Total (regional) diversity (*γ*) was partitioned into local *α* diversity (i.e., average diversity within a given sample) and among-sample *β* diversity (i.e., turnover diversity). Thus, *β* diversity was calculated as the total diversity minus the average local diversity (*β* = *γ*−*α*). The main advantage of additive partitioning, in characterizing the relationship between *α* and *β* components over the traditional multiplicative method (i.e., *γ* = *α*×*β*), is that the contribution of *α* and *β* to the total diversity can be directly calculated and compared [12], [40]. Consequently, when local diversity, on average, is lower than expected, we also expect high variation among samples (i.e., high *β* diversity) and vice versa.

As a first stage in this study, regional diversity was defined as the total number of TAUs found in the full collection of samples from the four sites. Alpha (*α*) diversity was the average number of TAUs within a transect (note that the terms diversity and number of TAUs are used interchangeably for convenience, but strictly speaking they are all the latter). Since we used a nested sampling design, samples at one scale are themselves composed of samples at a smaller scale. Hence, partitioning can be applied at all spatial scales and *γ* diversity can be partitioned into the diversity contributed by each scale. As such, *β*_1_ is regarded as the average diversity among transects, within stations; *β*_2_ is the average diversity between stations, within sites; and *β*_3_ is the average diversity among sites, within the region. Given our nested sampling design, these components can be calculated using the following equations:
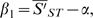
(1)

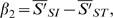
(2)

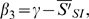
(3)where 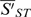 and 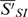 are the average number of TAUs within a station and a site, respectively. In combination, we express regional sampled diversity in terms of its *α* and *β* components, as:
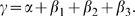
(4)Using the number of observed TAUs as a measure of regional diversity underestimates actual species diversity [42]; however, their consistent use across all spatial scales does not significantly alter the relationship among the diversity components [43].

Using a null model, all the individual coral colonies within the region were shuffled through individual-based randomization. This model is more powerful than the sample-based model in detecting departures between observed and null partitions because of intra-specific aggregation of individuals. Furthermore, the individual-based model overcomes the effects of abundance and sampling effort on diversity measures and comparisons (see Crist *et al.* [41] and Gotelli & Colwell [42] for further discussion). The model assumes that within the region an individual coral settles independent of locality and independent of the presence or absence of other corals. This approach preserves the original regional species-abundance distribution and the number of individuals within each transect. Following these constraints, we randomized all the individual coral colonies within the region and recorded the number of TAUs at each hierarchical level (i.e., *α*, 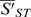 and 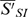). We then calculated the three components of *β* diversity (using equations 1–3), and repeated this procedure 1000 times to obtain a 95% confidence interval (CI) for each diversity component.

Deviations of the observed components from the null expectation indicate a non-random spatial distribution of TAUs. However, deviations of the mean diversity across the four tested sites can be the result of strong deviations within only some of the sites, whereas other sites may show diversity patterns similar to the expected overall null model. Alternatively, lack of deviations from the overall model may be the result of considerable negative deviations at some sites that are counterbalanced by high, positive deviations at other sites. Therefore, the observed diversity-component for each hierarchical level was calculated separately for each site and compared with those expected by the overall model that takes into account all the individual corals within the four sites. We then tested the relative contribution of each site to the observed regional diversity. In addition, the total diversity within each site was partitioned into the diversity contributed by each component within the site (*α*, *β*_1_ and *β*_2_). In these four independent analyses, *γ* diversity was defined as the total number of TAUs found in the full collection of samples within a site. The observed components were then compared with those expected by a null model that randomized all the individual corals within the tested site.

Multivariate analyses were conducted to examine differences in coral-community structure across the four sites (PRIMER®; [44]). The abundance of colonies within each TAU per transect was 4^th^-root transformed to reduce the influence of very dominant species (e.g., *Porites rus*; see ‘*Results*’). Thereafter, the Bray-Curtis similarity index was used to create a similarity matrix between transects, and differences among the sites were analyzed using non-metric multidimensional scaling (MDS) and one-way analysis of similarities (ANOSIM). The SIMPER (similarity percentages) routine was used to determine which TAUs made significant contributions to distinguishing differences among sites.

## Results

**Figure 3:**
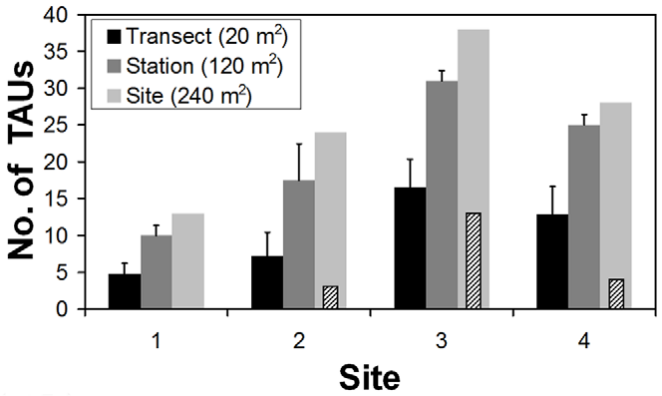
Comparison of number of coral TAUs (taxonomic units; mean + SD) at each site calculated per transect (n = 12), station (n = 2) and site (n = 1). Columns with diagonal lines show the number of ‘unique’ TAUs (i.e., TAUs that occur only at that site).

In total, 2,829 individual coral colonies were sampled, categorized into 46 TAUs ( = *γ* diversity). Diversity across the three tested spatial scales was highest at Site 3, followed by Site 4, Site 2 and Site 1 (Fig. 3). Site 3 also supported the highest number of ‘unique’ TAUs (13; i.e., TAUs occurring only at that site) and the highest number of ‘locally rare TAUs’ (11; i.e., TAUs found only in one transect within a site; Table 1). Site 1, on the other hand, did not support any unique TAUs and supported the least number of locally rare TAUs (3); in terms of community composition this site was nested within (i.e., it was a coral compositional of) the other three sites (Table 1).

**Table 1:** Inter-site comparisons showing the total number of coral TAUs (taxonomic units) at the study site (diagonal bold), where the numbers in parentheses are the frequency of locally rare TAUs (i.e., TAUs recorded only in one transect within a site).

Site	1	2	3	4
1	**13 (3)**	13	13	12
2		**24 (10)**	20	19
3			**38 (11)**	23
4				**28 (4)**

The number of TAUs that sites have in common is displayed above the diagonal.

**Figure 4:**
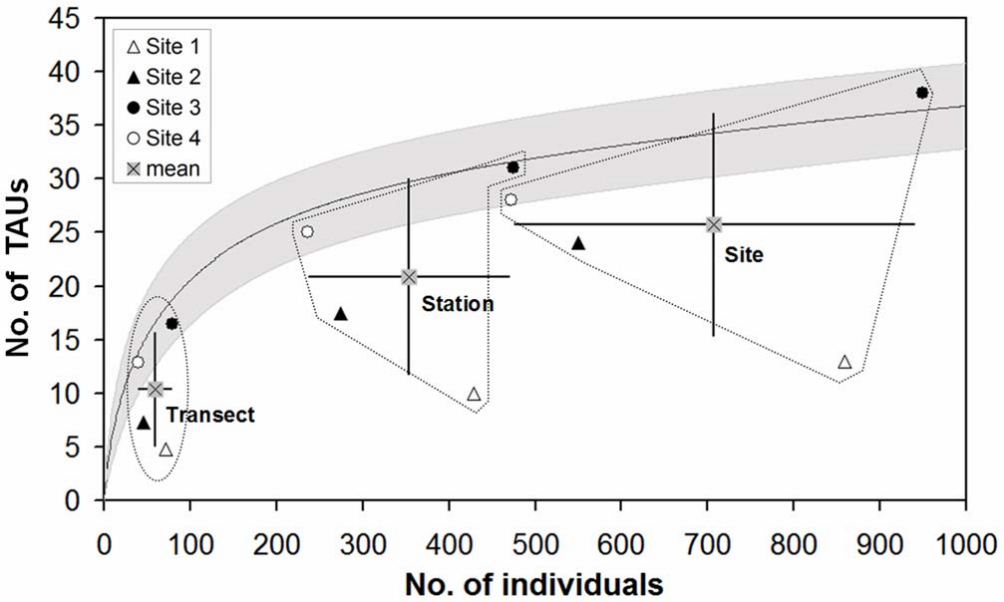
Observed number of coral TAUs (taxonomic units) found at each site across the three (hierarchical) sampling scales (i.e., transect, station and site) and expected number of TAUs generated by individual-based randomization (black line) including the 95% confidence interval envelop (shaded area). The expected curve is based on 2,829 individuals belonging to 46 TAUs, recorded within the four sites. The mean number of TAUs across the four sites for each hierarchical level is also presented as crosses. Horizontal and vertical error bars represent standard deviations.

The curve shown in Fig. 4 represents the expected number of TAUs per number of individuals, under the assumption that individual corals within the region settle independent of locality and independent of the presence or absence of other corals. In the three hierarchical scales the observed mean diversity was significantly lower than the expected diversity. The reason for these differences was primarily because diversity at Sites 1 and 2 was consistently lower than expected at all three scales. In contrast, diversity at Sites 3 and 4 did not deviate from the null expectation at any of the tested scales.

**Figure 5:**
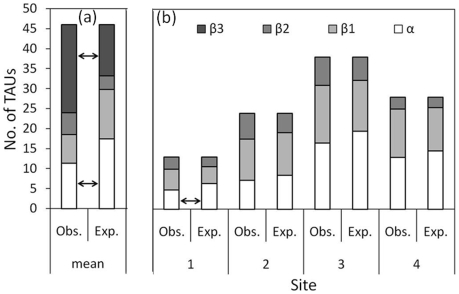
Additive partitioning of number of coral TAUs (taxonomic units) across the three (hierarchical) sampling scales (i.e., transect, station and site). The observed partitions (Obs.) are compared with the expected values (Exp.) as predicted by the overall (regional) null model, for (a) the mean across the four sites, and (b) separately for each site. Arrows indicate cases in which the observed diversity component differs (*p*<0.05) from the expected one.

Additive partitioning of the mean diversity across the four sites (Fig. 5a) showed that the (observed) *β*_3_ diversity component was significantly higher than the null expectation. Alternatively, *β*_1_ and *β*_2_ components were similar to those expected by the null model. As a result of the strong deviation of the *β*_3_ diversity component, the local *α* component (i.e., the contribution of the within-transect diversity relative to regional diversity) was significantly lower than expected. By applying the partitioning approach and a null model separately for each site (Fig. 5b), we found significant deviation from the expected partitioning at Site 1 only. This site supported significantly lower *α* diversity than expected.

**Figure 6:**
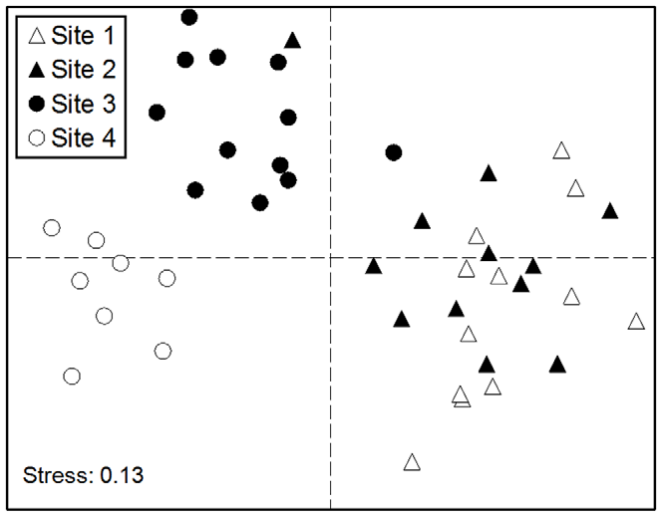
Multidimensional scaling (MDS) ordination based on Bray-Curtis similarities using 4^th^-root transformation, where each symbol represents a 20×1 m belt-transect.

The multivariate analysis showed that all pairwise comparisons among the four sites were statistically significant in terms of coral species composition, with the exception of Sites 1 and 2, which were similar (Fig. 6 and Table 2). SIMPER analysis showed that *P. rus* and the combined affect of *Fungia concinna*, *F. fungites*, *F. klunzingeri* and *Cycloseris* spp. contributed the most to the differences among sites (Table 3). *P. rus* was relatively abundant at Sites 1 and 2 and was rare at Sites 3 and 4, and the fungiids were relatively abundant at Site 3 and rare elsewhere.

**Table 2:** Analysis of similarity (ANOSIM) contrasts between sites, based on 9,999 permutations.

Contrasts	*R*	*p*
Site 1–Site 2	0.142	0.015
Site 1–Site 3	0.938	<0.0001*
Site 1–Site 4	0.996	<0.0001*
Site 2–Site 3	0.813	<0.0001*
Site 2–Site 4	0.949	<0.0001*
Site 3–Site 4	0.809	<0.0001*

The global *R* was 0.766 with *p*<0.0001. A pairwise comparison was considered significant (*) at the 5% level, if its *p*-value is below 0.0083, and not below 0.05 (using Bonferroni's adjustment).

**Table 3:** Dissimilarity percentages (SIMPER) of significant contrasts between sites.

TAU number	Species belonging to the TAU	Average abundance	Average abundance	Average dissimilarity	% contribution to dissimilarity	Cumulative % contribution
	Average dissimilarity = 75.8	Site 2	Site 3			
20	*Fungia concinna*, *Fungia fungites*, *Fungia klunzingeri*, *Cycloseris cyclolites*	0.08	22.08	17.3	22.82	22.82
44	*Porites rus*	20.42	8.33	13.57	17.9	40.73
42	*Porites cylindrica*	10.83	11	8.33	10.99	51.71
	Average dissimilarity = 78.32	Site 1	Site 3			
44	*Porites rus*	42.58	8.33	24.08	30.75	30.75
20	*Fungia concinna*, *Fungia fungites*, *Fungia klunzingeri*, *Cycloseris cyclolites*	0.17	22.08	13.84	17.68	48.42
42	*Porites cylindrica*	9.75	11	7.29	9.31	57.73
	Average dissimilarity = 91.3	Site 2	Site 4			
44	*Porites rus*	20.42	0.25	19.51	21.37	21.37
42	*Porites cylindrica*	10.83	0.5	11.79	12.92	34.29
38	*Pocillopora eydouxi*	0.08	8.42	10.26	11.23	45.52
22	*Galaxea astreata*	7.5	0.17	9.65	10.57	56.1
	Average dissimilarity = 92.4	Site 1	Site 4			
44	*Porites rus*	42.58	0.25	37.75	40.85	40.85
42	*Porites cylindrica*	9.75	0.5	9.09	9.83	50.68
	Average dissimilarity = 79.56	Site 3	Site 4			
20	*Fungia concinna*, *Fungia fungites*, *Fungia klunzingeri*, *Cycloseris cyclolites*	22.08	0.33	17.54	22.04	22.04
42	*Porites cylindrica*	11	0.5	8.41	10.57	32.61
38	*Pocillopora eydouxi*	0	8.42	7.47	9.39	42
44	*Porites rus*	8.33	0.25	6.24	7.84	49.84
39	*Pocillopora verrucosa*	3	3.58	3.4	4.27	54.11

TAU number	Species belonging to the TAU	Average abundance	Average abundance	Average dissimilarity	% contribution to dissimilarity	Cumulative % contribution
	Average dissimilarity = 75.8	Site 2	Site 3			
20	*Fungia concinna*, *Fungia fungites*, *Fungia klunzingeri*, *Cycloseris cyclolites*	0.08	22.08	17.3	22.82	22.82
44	*Porites rus*	20.42	8.33	13.57	17.9	40.73
42	*Porites cylindrica*	10.83	11	8.33	10.99	51.71
	Average dissimilarity = 78.32	Site 1	Site 3			
44	*Porites rus*	42.58	8.33	24.08	30.75	30.75
20	*Fungia concinna*, *Fungia fungites*, *Fungia klunzingeri*, *Cycloseris cyclolites*	0.17	22.08	13.84	17.68	48.42
42	*Porites cylindrica*	9.75	11	7.29	9.31	57.73

Lists of coral TAUs (taxonomic units) were truncated whenever the cumulative percentage was ≥50%.

## Discussion

This study shows that the mean diversity observed at the three hierarchical scales was consistently lower than expected by chance, under the assumption of a random distribution of TAUs around Zanzibar (Fig. 4). In addition, additive partitioning of mean diversity across the four sites (Fig. 5a) showed that *β*_3_ diversity (i.e., the contribution of the among-sites diversity) was significantly higher than expected by the null model. Thus, the local *α* diversity component was significantly lower than expected. Such deviations from the null expectations suggest that forces included in neutral theory [45], such as demographic stochasticity, may not be sufficient to explain diversity patterns around Zanzibar. Indeed, these results indicate that there are non-random processes interacting and disproportionately influencing spatial coral diversity patterns.

Our findings agree with a spatial study by Belmaker *et al.* [36], who sampled coral-dwelling fishes and their hosts (i.e., coral colonies from the genera *Stylophora*, *Pocillopora*, *Seriatopora* and *Acropora*) in three regions across the Indo-Pacific - Red Sea, Tanzania, and the Great Barrier Reef. Their coral-sampling design consisted of two hierarchical levels, both transects and sites, within each region. They found deviations at similar spatial scales in their three studied regions. However, in contrast with previous studies that partitioned only the mean diversity components within a region, the present study also separately tested the input of each site to the deviation from the overall (regional) null model. Indeed, using such examinations we found that deviations from the null expectations were the result of strong negative deviations only within Sites 1 and 2 (Fig. 4). On the other hand, Sites 3 and 4 showed diversity components similar to those expected by the overall null model.

Sites 1 and 2 are situated outside of any form of protection, whereas Sites 3 and 4 are situated within MPAs and experience a low degree of anthropogenic disturbance. Based on our data, however, we cannot explicitly identify the non-random processes that forced low diversity within Sites 1 and 2. Neither can we unequivocally suggest that the MPAs are causing high diversity within Sites 3 and 4. The latter sites may have been selected as MPAs because they supported high diversity. However, our findings do identify the sites where non-random processes reduce overall diversity, and highlight the need to further examine the mechanisms that reduce coral diversity at these sites. We show that these mechanisms interact on the among-sites scale (10's of kilometers), but also on the within transect-scale (i.e., ≤20 m) in Site 1. Over the larger scale, these mechanisms can range from dispersal-limitation to differential survival among the sites. However, in Site 1, there are mechanisms that prevent the success of some species in specific locations and reduce local *α* diversity. These mechanisms can range from interspecific interactions to human disturbances that interact on that scale.

The MDS ordination (Fig. 6) and ANOSIM (Table 2) supported these results by showing that each of the studied sites was different in terms of coral species composition. *P. rus* was found to be relatively abundant at Sites 1 and 2 (43% and 20%, respectively), and was shown to be among the most important taxa contributing to the differences among sites (Table 3). *P. rus* formed large monospecific stands at both sites, considerably reducing local diversity potential. In theory, interspecific interactions might exclude species from some habitats and force others into a dominant role, but there is little evidence supporting this mechanism in determining local coral composition [5], [46]–[47]. Although competitive interactions between corals are prolific, there is no evidence of a consistent hierarchical pattern, whereby one species always wins [48]–[49]. In other words, while among coral-species competition exists on coral reefs, it does not alone drive local coral species diversity [5]. On the other hand, habitat selection through differential propagule settlement [50]–[52], biotic factors (e.g., fish predation; [53]), or adaptation to different abiotic environments [e.g.], [11, 54], are all known to play a role in habitat specialization. *P. rus* is a fast-growing species that covers large areas of reefs and contributes greatly to the coral coverage. Therefore, even though there are processes that interact within Sites 1 and 2 leading to a reduced local diversity, this is not expressed in reduced coral coverage.

Inter-site comparisons showed that Site 1, in contrast to the other sites, did not support any unique TAUs (i.e., TAUs occurring only at that site; see Fig. 3), and showed the fewest locally rare TAUs (i.e., TAUs recorded only in one transect within a site; see Table 1). Indeed, in terms of species composition this site was nested (a subset) within the other three sites (Table 1). Similar findings have been reported for the Great Barrier Reef [55], and Micronesia [56], where highly stressed sites support only widespread, regionally dominant species. Site 2, which is also a non-protected site, supported unique TAUs and showed a much larger number of locally rare TAUs than Site 1. In addition, 50% of the locally rare TAUs found at Site 2 were rare elsewhere, whereas none of the three locally rare TAUs found at Site 1 were rare at the other sites. Species with restricted distributions are known to be highly susceptible to habitat degradation [57], and thus are important criteria for site selection by conservationists [58]. Therefore, with regard to regional-diversity conservation, and based on comparison of unique TAUs and locally rare TAUs between Sites 1 and 2, we strongly recommend that Site 2 (Bawe) be declared a MPA. Regarding Sites 3 and 4, our results unreservedly support their declared status as MPAs.

To the best of our knowledge, this is the first study to use additive partitioning of biodiversity in order to study spatial patterns of stony-coral communities. The additive partitioning approach has indicated that there are non-random processes governing the observed coral diversity patterns around Zanzibar. Indeed, this approach not only helped to identify a site where non-random processes interact and disproportionately differentiate the overall diversity, but it also enabled us to highlight the appropriate spatial boundaries for studying the mechanisms that may decrease coral diversity. Based on our results, at a time in history when understanding the underlying mechanisms that regulate the spatial distribution of corals is urgent, we suggest that further work needs to be done in order to understand the processes that may be interacting locally, especially at Site 1, to reduce coral diversity.

## Supporting Information

Table S1 List of coral TAUs (taxonomic units), including number of individuals observed in the survey and species included within each TAU.
(0.07 MB DOC)
