# Supplementary material for: Diversity Partitioning of Stony Corals Across Multiple Spatial Scales Around Zanzibar Island, Tanzania
Source: PLoS One. 2010 Mar 29;5(3):e9941. doi: 10.1371/journal.pone.0009941 (PMC2847908; doi:10.1371/journal.pone.0009941)
Supplement: Table S1 — List of coral TAUs (taxonomic units), including number of individuals observed in the survey and species included within each TAU. (0.07 MB DOC) [file pone.0009941.s001.doc]

**Table S1**. List of coral TAUs (taxonomic units), including number of individuals observed in the survey and species included within each TAU.

| TAU number | No. of individuals observed | Species belonging to the TAU | | |  |
| --- | --- | --- | --- | --- | --- |
| 1 | 1 | *Acanthastrea hemprichii* | *Acanthastrea lordhowensis* | *Plesiastrea versipora* |  |
| 2 | 1 | *Acropora anthocercis* |  |  |  |
| 3 | 28 | *Acropora austera* | *Acropora formosa* | *Acropora forskali* |  |
|  |  | *Acropora grandis* | *Acropora caespitose* |  |  |
| 4 | 27 | *Acropora cerealis* | *Acropora donei* | *Acropora secale* |  |
|  |  | *Acropora subulata* | *Acropora digitifera* |  |  |
| 5 | 1 | *Acropora clathrata* | *Acropora glauca* |  |  |
| 6 | 91 | *Acropora corymbose* | *Acropora gemmifera* | *Acropora humilis* |  |
|  |  | *Acropora monticulosa* |  |  |  |
| 7 | 47 | *Acropora divaricata* | *Acropora polystoma* |  |  |
|  |  | *Acropora latistella* | *Acropora nasuta* | *Acropora selago* |  |
| 8 | 77 | *Acropora tenuis* | *Acropora valida* | *Acropora cytherea* |  |
|  |  | *Acropora hyacinthus* |  |  |  |
| 9 | 16 | *Acropora nobilis* |  |  |  |
| 10 | 35 | *Acropora palifera* |  |  |  |
| 11 | 3 | *Astreopora expansa* |  |  |  |
| 12 | 1 | *Caulastrea tumida* |  |  |  |
| 13 | 5 | *Cyphastrea chalcidicum* | *Cyphastrea serailia* | *Diploastrea heliopora* | |
| 14 | 2 | *Echinophyllia orpheensis* |  |  |  |
| 15 | 33 | *Echinopora hirsutissima* | *Montastrea magnistellata* |  |  |
| 16 | 20 | *Echinopora lamellosa* |  |  |  |
| 17 | 18 | *Favia favus* | *Favia lizardensis* | *Favia speciosa* |  |
|  |  | *Favia veroni* | *Favia laxa* |  |  |
| 18 | 3 | *Favia stelligera* |  |  |  |
| 19 | 3 | *Favites abdita* | *Favites complanata* | *Favites halicora* |  |
| 20 | 272 | *Fungia concinna* | *Fungia fungites* | *Fungia klunzingeri* |  |
|  |  | *Cycloseris cyclolitics* |  |  |  |

| TAU number | No. of individuals observed | Species belonging to the TAU | | |
| --- | --- | --- | --- | --- |
| 21 | 25 | *Fungia paumotensis* | *Fungia scutaria* |  |
| 22 | 232 | *Galaxea astreata* |  |  |
| 23 | 29 | *Galaxea fascicularis* |  |  |
| 24 | 2 | *Gardineroseris planulata* |  |  |
| 25 | 2 | *Goniastrea pectinata* |  |  |
| 26 | 21 | *Goniastrea retiformis* |  |  |
| 27 | 1 | *Halomitra pileus* |  |  |
| 28 | 3 | *Herpolitha weberi* |  |  |
| 29 | 1 | *Hydnophora microconos* |  |  |
| 30 | 7 | *Lobophyllia hemprichii* |  |  |
| 31 | 22 | *Millepora intricata* | *Millepora tenella* |  |
| 32 | 5 | *Millepora platyphylla* |  |  |
| 33 | 1 | *Montipora digitata* | *Montipora informis* | *Montipora verrucosa* |
| 34 | 1 | *Physogyra lichtensteini* |  |  |
| 35 | 1 | *Platygyra crosslandi* |  |  |
| 36 | 21 | *Platygyra daedalea* | *Platygyra sinensis* | *Platygyra lamellina* |
|  |  | *Goniastrea australensis* |  |  |
| 37 | 67 | *Pocillopora darmicornis* |  |  |
| 38 | 102 | *Pocillopora eydouxi* |  |  |
| 39 | 80 | *Pocillopora verrucosa* |  |  |
| 40 | 1 | *Polyphilla talpina* |  |  |
| 41 | 5 | *Porites annae* | *Porites nigrescens* | *Porites profundus* |
| 42 | 385 | *Porites cylindrica* |  |  |
| 43 | 197 | *Porites lobata* | *Porites lutea* |  |
| 44 | 859 | *Porites rus* |  |  |
| 45 | 22 | *Seriatopora aculeata* | *Seriatopora hystrix* |  |
| 46 | 54 | *Stylophora pistillata* |  |  |
